# Therapeutic effect of curcumin nanoemulsion on cystic echinococcosis in BALB/c mice: a computerized tomography (CT) scan and histopathologic study evaluation

**DOI:** 10.1186/s12906-024-04451-z

**Published:** 2024-04-04

**Authors:** Mohamad Ghanimatdan, Seyed Mahmoud Sadjjadi, Fattaneh Mikaeili, Aref Teimouri, Seyed Hamed Jafari, Amin Derakhshanfar, Saeideh Hashemi-Hafshejani

**Affiliations:** 1 Department of Parasitology and Mycology, School of Medicine, Shiraz University of Medical Sciences, Shiraz, Iran; 2 Department of Radiology, School of Medicine, Shiraz University of Medical Sciences, Shiraz, Iran; 3 Department of Comparative Biomedical Sciences, School of Advanced Medical Sciences and Technologies, Shiraz University of Medical Sciences, Shiraz, Iran; 4 Department of Parasitology and Mycology, School of Medicine, Iran University of Medical Sciences, Tehran, Iran

**Keywords:** Therapeutic efficacy, In vivo, Curcumin nanoemulsion, *Echinococcus granulosus*, Cystic echinococcosis, CT scan, Calcification

## Abstract

**Background:**

This study aimed to determine the therapeutic efficacy of curcumin nanoemulsion (CUR-NE) in mice infected with *Echinococcus granulosus* sensu stricto protoscoleces.

**Methods:**

Forty-two inbred BALB/c mice were divided into seven groups of six animals each. Six groups were inoculated intra-peritoneally with 1500 viable *E. granulosus* protoscoleces, followed for six months and used as infected groups. The infected groups were named as: CEI1 to CEI6 accordingly. The 7th group was not inoculated and was named cystic echinococcosis noninfected group (CENI7). CEI1 and CEI2 groups received 40 mg/kg/day and 20 mg/kg/day curcumin nanoemulsion (CUR-NE), respectively. CEI3 received nanoemulsion without curcumin (NE-no CUR), CEI4 received curcumin suspension (CUR-S) 40 mg/kg/day, CEI5 received albendazole 150 mg/kg/day and CEI6 received sterile phosphate-buffered saline (PBS). CENI7 group received CUR-NE 40 mg/kg/day. Drugs administration was started after six months post-inoculations of protoscoleces and continued for 60 days in all groups. The secondary CE cyst area was evaluated by computed tomography (CT) scan for each mouse before treatment and on the days 30 and 60 post-treatment. The CT scan measurement results were compared before and after treatment. After the euthanasia of the mice on the 60th day, the cyst area was also measured after autopsy and, the histopathological changes of the secondary cysts for each group were observed. The therapeutic efficacy of CUR-NE in infected groups was evaluated by two methods: CT scan and autopsied cyst measurements.

**Results:**

Septal calcification in three groups of infected mice (CEI1, CEI2, and CEI4) was revealed by CT scan. The therapeutic efficacy of CUR-NE 40 mg/kg/day (CEI1 group) was 24.6 ± 26.89% by CT scan measurement and 55.16 ± 32.37% by autopsied cysts measurements. The extensive destructive effects of CUR-NE 40 mg/kg/day (CEI1 group) on the wall layers of secondary CE cysts were confirmed by histopathology.

**Conclusion:**

The current study demonstrated a significant therapeutic effect of CUR-NE (40 mg/kg/day) on secondary CE cysts in BALB/c mice. An apparent septal calcification of several cysts revealed by CT scan and the destructive effect on CE cysts observed in histopathology are two critical key factors that suggest curcumin nanoemulsion could be a potential treatment for cystic echinococcosis.

## Introduction

Cystic echinococcosis (CE) is a zoonotic disease caused by the larval stage of *Echinococcus granulosus* sensu lato (*E. granulosus s.l*) [1]. The World Health Organization (WHO) has classified CE as a neglected tropical disease [2]. CE is endemic in Africa, Asia, Europe, South America, Australia and New Zealand. The Eastern Mediterranean region, including Iran, is considered an important endemic region of CE [3].

*E. granulosus* typically infects herbivorous mammals, including sheep, goats, cattle, and camels, which serve as intermediate hosts. The adult tapeworm resides in the small intestine of canids, including dogs, wolves, foxes, and jackals (definitive hosts) that sheds eggs with faeces. Humans can also be infected accidentally by ingesting food or water contaminated with eggs or through direct contact with an infected dog [4]. Following ingestion of *E. granulosus* eggs, CE cysts may develop anywhere in the human body, mainly in the liver and lungs [5]. Imaging studies have shown that CE cysts can grow 1 to 50 millimeters (mm) yearly or persist without alteration for a long time [5]. Clinical symptoms which vary depending on the organ involved, usually occur following excessive growth of cysts that enlarge the abdomen and sometime rupture of the cysts may cause anaphylactic shock [6]. Based on phylogenetic studies, there are ten recognized genotypes (G1-G10) of *E. granulosus*, including *E. granulosus* sensu stricto (G1- G3), *E. equinus* (G4), *E. ortleppi* (G5), and *E. canadensis* (G6- G8 and G10) [7].

CE presents a significant challenge in terms of treatment. While surgery was the only available treatment option for CE for a long time, it is no longer the primary or optimal treatment option in all cases [8]. Surgical treatment is preserved for complicated cysts (ruptured, perforated, compression on vital organs and vessels, secondary infection), large cysts containing daughter cysts, and superficial cysts [8, 9]. Otherwise, alternative methods, such as the use of anthelmintic medications like albendazole (ABZ) and praziquantel or minimally invasive surgical procedures such as puncture (P), aspiration (A), instillation (I), and re-aspiration (R) PAIR method and also watch and wait, should be taken into account [8, 10].

ABZ (the first-line anthelmintic chemotherapy for CE) works by inhibiting microtubule polymerization in the parasite, which leads to the disruption of cytoplasmic microtubules and the inhibition of glucose uptake. This leads to the depletion of energy reserves in the parasite, and its death [11]. Studies have indicated that ABZ can permeate the cyst membrane and attain therapeutic concentrations within the cyst [12]. The efficacy of ABZ is hindered by its low water solubility (which results in low bioavailability), side effects, and high cost. To compensate for this therapeutic shortfall, it is crucial to discover a new medication that can treat CE with high efficacy and minimal side effects [13, 14].

Over the last decade, traditional natural medicines have been extensively studied in pharmaceutical research due to their inherent biodegradability and relatively nontoxic nature for various organisms [15, 16]. To date, approximately fifty-eight different plant species have been evaluated against *E. granulosus* protoscoleces (*Zataria multiflora* extract was used most extensively, followed by *Nigella sativa*, *Berberis vulgaris*, *Zingiber officinale* (ginger), and *Allium sativum*) [17]. Curcumin (CUR), also called diferuloylmethane, is the main natural polyphenol found in the rhizome of *Curcuma longa* (turmeric) and other *Curcuma* spp [18]. The safety, tolerability, and nontoxicity of CUR has been extensively verified, even at high doses [19]. Despite their potential benefits, the hydrophobic nature of these derivatives, coupled with their poor water solubility, chemical instability, low bioavailability, and rapid metabolism, pose significant challenges to their application [20, 21]. Various formulations have been described to enhance the bioavailability of CUR. Among these formulations, nanoemulsions (NEs) have been widely developed to improve CUR solubility and bioavailability [22, 23]. The size of the NE droplets ranges from 10 to 600 nm. Applying NEs can result in improved medication effectiveness and decreased occurrence of detrimental side effects and toxic reactions [24].

To the best of the author’s knowledge, although various in vitro studies have examined the protoscolicidal activity of CUR and CUR-NE against *E. granulosus* protoscoleces [25–28], the in vivo effects of CUR-NE on secondary CE have not been determined, so far. Some in vivo CE study challenges (inability to monitor secondary cysts) can be resolved by using CT scan modality, which accurately measures secondary cyst dimensions in each mouse before, during and after treatment. It also identifies calcification effectively. Therefore, the present study was conducted to assess the therapeutic efficacy of CUR-NE on secondary CE cysts in inbred BALB/c mice using CT scan. *E. granulosus* sensu stricto (G1-G3) is the predominant genotype in humans and livestock in Iran [29–33]. Therefore, in the current study, the G1 genotype was used for the protocol. Moreover, the cytotoxicity of CUR and CUR-NE was evaluated in the HeLa cell line.

## Material and method

### Compounds

Curcumin (CAS-No: 458-37-7; purity: ≥80%), ABZ (CAS-No: 54965-21-8; purity: ≥98%), eosin powder, and soybean oil were purchased from Sigma-Aldrich, St. Louis, MO, USA. Furthermore, ethanol, polysorbates of tween 80 and tween 85 and sodium chloride (Merck, Germany) were also used in the present study.

### Preparation of the curcumin nanoemulsion

CUR-NE (2000 µg/ml) was successfully prepared using spontaneous emulsification method in the Department of Parasitology and Mycology, Shiraz University of Medical Sciences, Iran. Briefly, curcumin was dissolved in soybean oil (oil phase) by gradually adding a mixture of Tween 80 and 85 (surfactant), ethanol (co-surfactant), and distilled water with constant stirring by a magnet [28]. Chemicals and medications were stored at 4 °C.

### Cytotoxicity evaluation of curcumin compounds

The evaluation was performed using cell culture techniques as follows: the HeLa cell line was obtained from the Parasitology and Mycology Department, Shiraz University of Medical Sciences, Iran. The cells were cultured in Dulbecco’s Modified Eagle’s Medium (DMEM) (Sigma, USA) supplemented with 10% heat-inactivated fetal bovine serum (FBS) and 1% (v/v) penicillin-streptomycin and incubated at 37˚C in humidified 5% CO2 and 95% air atmosphere. The cytotoxicity of different concentrations of CUR-NE, curcumin suspension (CUR-S), and nanoemulsion without curcumin (NE-no CUR) on HeLa cells was assessed by a 3-(4,5-dimethylthiazol2-yl)-2,5-diphenyltetrazolium bromide (MTT) assay. The final concentrations of compounds were 125–2000 µg/ml. briefly, 3 × 10^4^ cells/well were treated with various concentrations of CUR-NE, CUR-S, and NE-no CUR. The Plates were incubated for 24 hours at 37˚C in 5% CO2. Three wells were considered blanks, which only contained culture medium. MTT reagent (5 mg/ml) was added to each well, and the plate was incubated in a dark room at 37˚C for four h. The optical density (OD) was read at a wavelength of 570 nm using an ELISA reader (Bio-Rad). All experiments were repeated at least three times. The percentage of cell viability and cytotoxicity was determined for each compound concentration by applying the following formula [34]:

$${\rm{Cell}}\,{\rm{viability}}\,\left( \% \right) = {{{\rm{OD}}\left( {{\rm{Test}}} \right) - {\rm{OD}}\left( {{\rm{Blank}}} \right)} \over {{\rm{OD}}\left( {{\rm{Control}}} \right) - {\rm{OD}}\left( {{\rm{Blank}}} \right)}} \times 100$$

$$\text{C}\text{y}\text{t}\text{o}\text{t}\text{o}\text{x}\text{i}\text{c}\text{i}\text{t}\text{y} \left(\text{\%}\right)=100-\text{V}\text{i}\text{a}\text{b}\text{i}\text{l}\text{i}\text{t}\text{y} \left(\text{\%}\right)$$

### In vivo toxicity of CUR-NE

To evaluate the in vivo toxicity of the highest safe concentration of CUR-NE, a total of six cystic echinococcosis noninfected BALB/c mice (CENI7) were used. They received the highest safe concentration of CUR-NE orally for 60 days. At the end of treatment, liver tissue histopathology was assessed to identify any abnormal alterations.

### Collection of *E. granulosus* protoscoleces

The CE cysts were obtained from the livers of naturally infected sheep slaughtered at Shiraz slaughterhouse, Fars province, Iran. The CE cyst fluid and protoscoleces (PSCs) were collected under sterile conditions and transferred into 50 mL Falcon tubes. After 20 min of sedimentation, the supernatant was discarded, and PSCs were washed three times with sterile phosphate-buffered saline (PBS). The viability of PSCs was assessed using vital staining with 0.1% eosin, and their movement was observed under a microscope [35, 36]. PSCs with a viability of more than 90% were used to induce secondary CE in BALB/c mice. The PSCs were stored in sterile PBS at a temperature of 4 °C until use.

### Genotyping

DNA was extracted from *E. granulosus* PSCs using a commercial kit (ViraGen, Tehran, Iran). The cox1 gene of *E. granulosus* was amplified using specific primers JB3 and JB4.5 through polymerase chain reaction (PCR) [37], and the PCR products were sequenced with the Sanger method.

### In vivo effect of CUR-NE on cystic echinococcosis

#### Induction of secondary CE in mice

Forty-two inbred female BALB/c mice with an average age of six weeks and an average weight of 25 ± 5 g were used as laboratory animals. To induce secondary CE in mice, thirty-six mice were inoculated intraperitoneally (IP) with 1500 *E. granulosus* s.s. PSCs. The remaining six mice were kept under the same conditions but were not inoculated with PSCs at all. The mice were housed in appropriate environmental conditions and had free access to water and food (*Ad libitum*). The mice were followed daily for six months until the signs of secondary CE cysts were observed by increasing their abdomen size as well as CT scan monitoring.

#### Primary evaluation (before treatment) of secondary CE cysts in mice

To evaluate the development of secondary CE cysts in mice, an initial CT scan examination was conducted on all CE-infected BALB/c mice six months post inoculation of PSCs. The mice were first anesthetized with ketamine and xylazine at a dosage of 100 mg/kg and 10 mg/kg, respectively. Following the imaging procedure, the qualitative parameters of secondary cysts, such as calcification [38], and the quantitative parameters, including cyst size and area in each mouse, were assessed and recorded.

All animal experiments were conducted in accordance with the guidelines of the Ethics Committee of Shiraz University of Medical Sciences, Iran (IR.SUMS.AEC.1401.114).

#### Treatment of CE-infected BALB/c mice

Six months post-inoculation of mice with PSCs; the treatment experiment was performed on thirty-six BALB/c mice with CE cysts. The mice were randomly divided into six groups containing six animals/group.

1. Treatment group 1: Cystic echinococcosis infected group 1 (CEI1) received curcumin nanoemulsion (CUR-NE) (40 mg/kg/day).
2. Treatment group 2: CEI2 received CUR-NE (20 mg/kg/day).
3. Treatment group 3: CEI3 received nanoemulsion without curcumin (NE-no CUR).
4. Treatment group 4: CEI4 received curcumin suspension (CUR-S) (40 mg/kg/day).
5. Positive control group: CEI5 received ABZ (150 mg/kg/day).
6. Negative control group: CEI6 received sterile PBS.

The treatment was performed in all groups using oral gavage for 60 days with similar daily conditions and at the same time. Each group of mice was numbered and monitored until the end of the experiments. The weights of the mice were measured before treatment and 60 days post treatment protocol. The highest safe concentration of CUR-NE for treatment of infected BALB/c mice was selected based on its toxicity. To achieve this, this concentration was assessed for its cell toxicity and in vivo toxicity. At the 1000 and 1500 µg/ml, CUR-NE showed cytotoxicity in less than 50% of the HeLa cells, while the IC50 of CUR-NE was found to be 1495.66 µg/ml. Therefore, the 1000 µg/ml concentration was determined as the threshold for safety and utilized for further in vivo toxicity assessment. This concentration of CUR-NE did not cause noticeable histopathological alterations in the livers of non-infected mice. Therefore, the 1000 µg/ml was determined as the highest safe concentration for the treatment of infected BALB/c mice.

Most in vivo studies of cystic echinococcosis used gavage to deliver the agents orally. Therefore, oral administration was chosen in this study.

#### Assessment of the therapeutic effect of CUR-NE on CE in mice

**Fig. 1:**
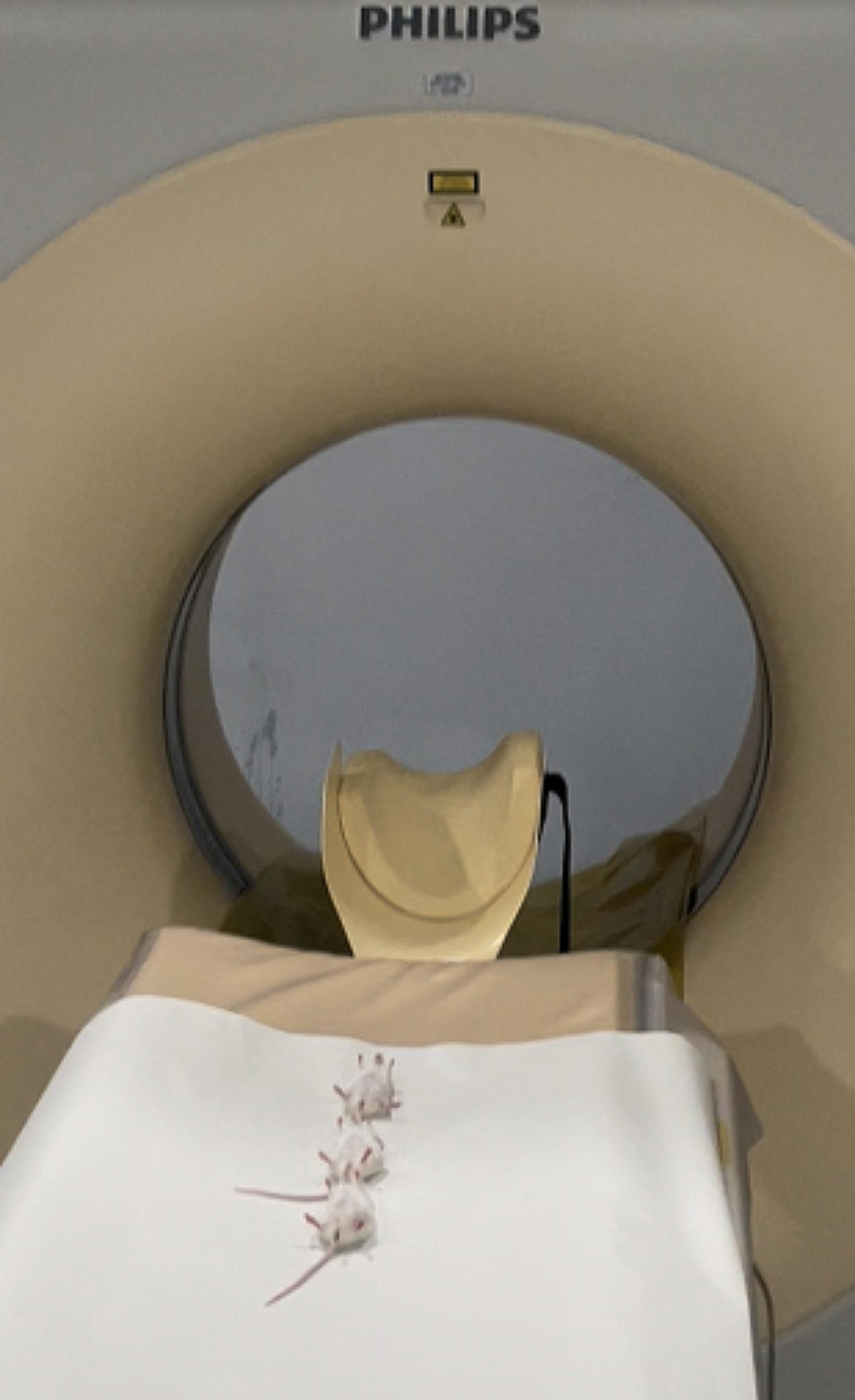
Each mouse was scanned thrice by CT (before treatment, 30 and 60 days after treatment)

Cyst dimensions were measured using CT scan (Philips. The Netherlands) before treatment and on the days 30 and 60 post treatment to assess the therapeutic efficacy for each mouse in all groups (Fig. 1). The mice were anesthetized with ketamine and xylazine, and then a CT scan was performed to measure the qualitative and quantitative parameters of secondary CE cysts.

The modified following formula was used for therapeutic efficacy determination [39]:

$${\rm{Therapeutic}}\,{\rm{efficacy}}\left( \% \right) = {{{a_1} - {a_2}} \over {{a_1}}} \times 100$$

In the formula, a_1_ represents the cyst area before treatment, while a_2_ represents the cyst area post treatment in each mouse.

At the end of treatment, the mice were euthanized by administration of CO_2_ gas. Subsequently, their CE cysts were removed from the peritoneal cavity. The secondary cysts were also photographed using a digital camera and later used for measuring their dimensions by Digimizer software (v6.0). In brief, a scale is set on the ruler, then the cyst boundary is defined, and its area is measured by software. The dimensions of all cysts isolated from the peritoneal cavity of mice with CE were measured using Digimizer software. The same procedure was used for all of the secondary cysts and they were measured with the same scale.

Therapeutic efficacy was also determined using the mean cyst area after treatment measured by Digimizer software. For this, the cyst area in mice in the treatment groups and the mean cyst area in mice in the negative control group (NC) were used in the following formula [39]: This formula uses ${\text{a}}_{\text{N}\text{C}}$ to represent the mean cyst area in the NC group and ${\text{a}}_{\text{T}}$ to represent the cyst area in treated mice.

$${\rm{Therapeutic}}\,{\rm{efficacy}}\left( \% \right) = {{{a_{NC}} - {a_T}} \over {{a_{NC}}}} \times 100$$

#### Histopathological evaluation of the cysts

The CE cysts removed from the mice were preserved in 10% buffered formalin and transported to the histopathology laboratory. After 24 h, the samples were replaced with new 10% buffered formalin. After fixation, dehydration was carried out using serial of ethanol concentrations, including 70%, 90%, 96%, and pure 100% ethanol. The samples were embedded in paraffin, and blocks were subsequently created. Serial sections with a thickness of 5 micrometers were prepared and stained with hematoxylin and eosin (H&E) [40]. The histopathological changes in cyst layers were assessed using light microscopy.

### Statistical analysis

The quantitative data, including the cyst area measurements by CT scan before and after treatment, the autopsied cyst measurements after treatment, the weight of mice before and after treatment, and qualitative data such as calcification, were transferred to a data sheet and analyzed by SPSS version 21 software. The data were normalized using Kolmogorov‒Smirnov and Shapiro‒Wilk tests. After confirming the normality of the data, Student’s t test was used to compare the statistical differences within the groups, and one-way ANOVA was used to compare the differences between groups. Additionally, the chi-square test was used to compare the qualitative data.

## Results

### The cytotoxicity of curcumin compounds

**Fig. 2:**
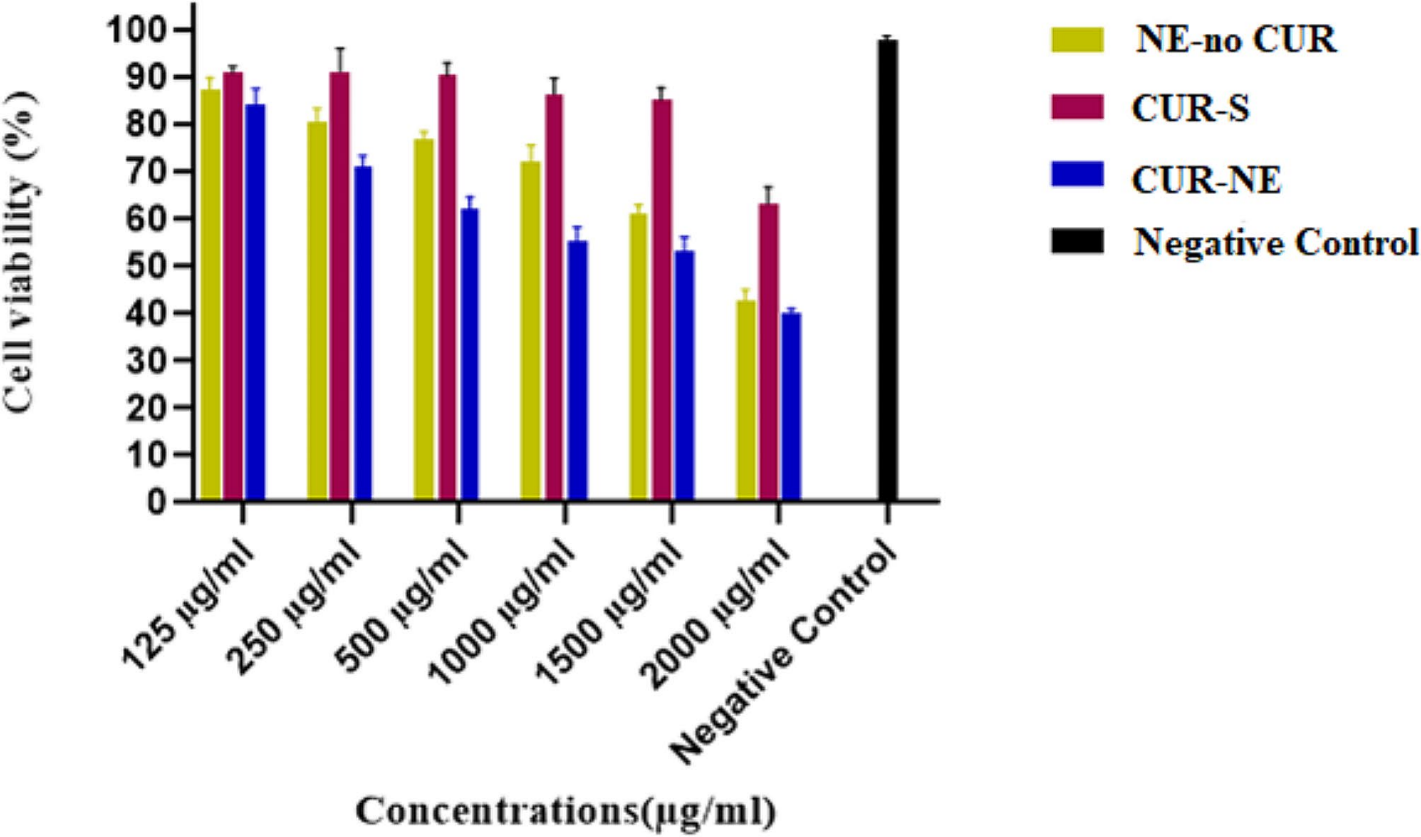
The viability percentage of HeLa cells after 24 h of treatment with different components. CUR-NE (blue bar), CUR-S (red bar), NE-no CUR (yellow bar) and negative control (black bar)

The percentage of viable HeLa cells was assessed at different concentrations of CUR-NE, CUR-S and NE-no CUR (125–2000 µg/ml) for 24 h (Fig. 2). Based on these findings, the 2000 µg/ml concentration of all three components showed the maximum cytotoxic effect on HeLa cells. Moreover, the concentration of all components and the viability of HeLa cells had an inverse relationship. The viability of HeLa cells after treatment with all concentrations of CUR-S was greater than 60%. The viability rate of the cells was higher than 50% when treated with CUR-NE and NE-no CUR up to a concentration of 1500 µg/ml. While at a concentration of 2000 µg/ml, the cell viability percentage dropped below 50%.

The IC50 values of CUR-NE, CUR-S and NE-no CUR were estimated to be 1495.66 µg/ml, 3222.7 µg/ml, and 1832.53 µg/ml, respectively.

### The in vivo toxicity of CUR-NE

**Fig. 3:**
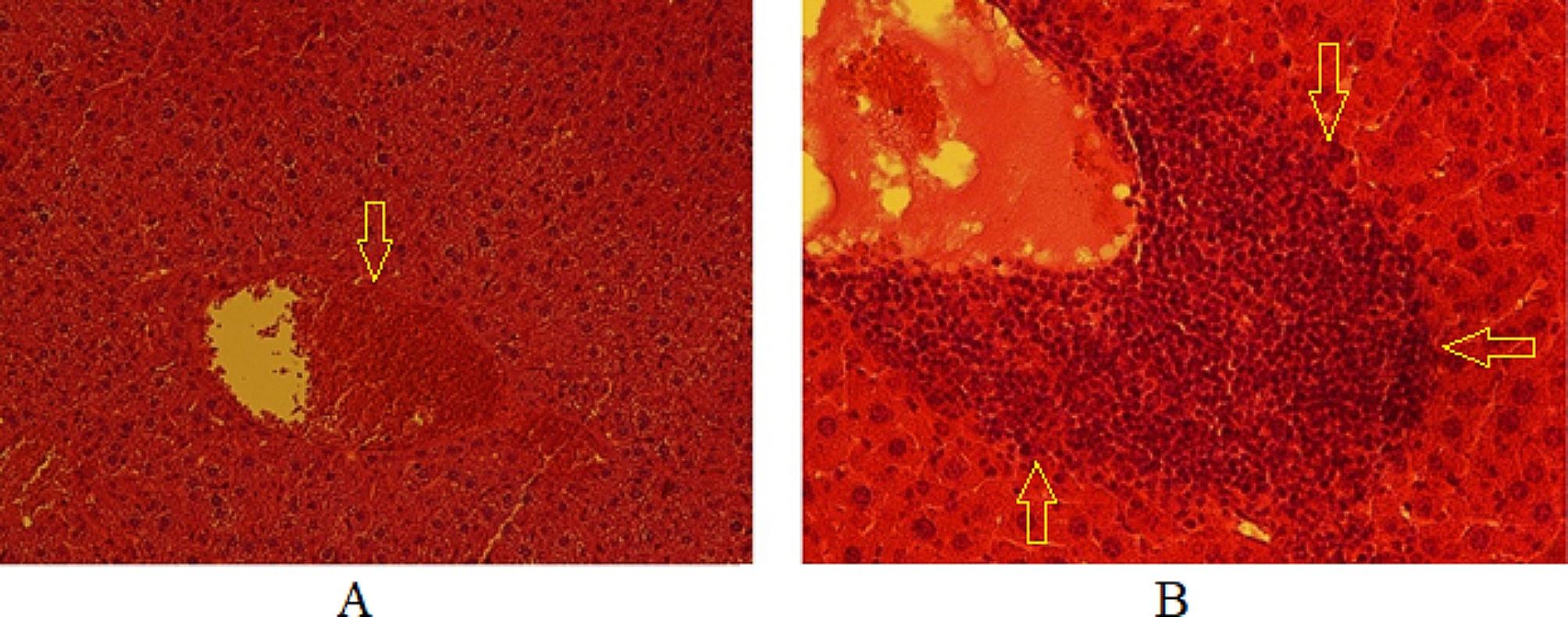
Histopathological sections of liver tissue in cystic echinococcosis noninfected BALB/c mice (CENI7) correspond to a CUR-NE (40 mg/kg/day) treated group. **A**: Dilation of the central vein and hyperemia x100, H&E; **B**: Focal infiltration of lymphocytes around the central vein x200, H&E

The highest safe concentration of CUR-NE (1000 µg/ml) showed less than 50% cytotoxicity, upon administering 0.5 ml of 2000 µg/ml CUR-NE to non-infected BALB/c mice over a 60 day period, their liver tissues showed no noticeable histopathological deviations. No significant pathological alteration were observed in non-infected mice that received CUR-NE (40 mg/kg/day), except for focal infiltration of lymphocytes around the central vein and dilation of the central vein (Fig. 3).

### Echinococcus granulosus genotype

For all samples in the present study, a 444-base pair (bp) fragment of the cox1 gene of *E. granulosus* was successfully amplified by polymerase chain reaction (PCR) method. Based on the sequence alignment analysis of the cox1 gene, all *E. granulosus* isolates were identified as *E. granulosus* sensu stricto (G1-G3) isolates. The sequences obtained from this study have been deposited in the GenBank database with accession numbers OQ525794, OQ520283, OQ520281, and OQ520278.

### Weight differences in mice before and after treatment

The treatment results demonstrated a lower mean weight of mice in the CEI1 and CEI4 groups, while this trend was not observed in the other groups. The weight of mice before treatment and 60 days post treatment showed a significant difference between groups (*P* = 0.002). The results of the post hoc Tukey HSD test revealed a significant difference in the weight of mice before and after treatment in the CEI1 and negative control groups (*P* = 0.000), as well as the difference in the weight of mice before and after treatment in the CEI3 and CEI4 groups (*P* = 0.023) (Table 1).

**Table 1:** Mean ± SD weight of mice before and 60 days after treatment in different groups

Groups	n	Mean ± SD weight of mice before treatment	Mean ± SD weight of mice after treatment
CEI1: treated with curcumin nanoemulsion (CUR-NE) (40 mg/kg/day)	6	31.2 ± 2.58	29.4 ± 2.07
CEI2: treated with CUR-NE (20 mg/kg/day)	6	33.80 ± 3.03	34.00 ± 1.73
CEI3: treated with NE without CUR (NE-no CUR)	6	37.4 ± 3.91	38.8 ± 5.11
CEI4: treated with curcumin suspension (CUR-S) (40 mg/kg/day)	6	33.8 ± 3.7	32.4 ± 2.19
CEI5: treated with ABZ (150 mg/kg/day) (PC^1^)	6	34.2 ± 2.86	35.00 ± 2.00
CEI6: received sterile PBS (NC^2^)	4	30.67 ± 3.67	34.33 ± 3.14

ABZ: albendazole; PBS: phosphate-buffered saline

^1^Positive control; ^2^Negative control

### Assessment of the therapeutic effect of CUR-NE on CE in mice

#### Determination of the therapeutic efficacy of CUR-NE using CT scan results

**Fig. 4:**
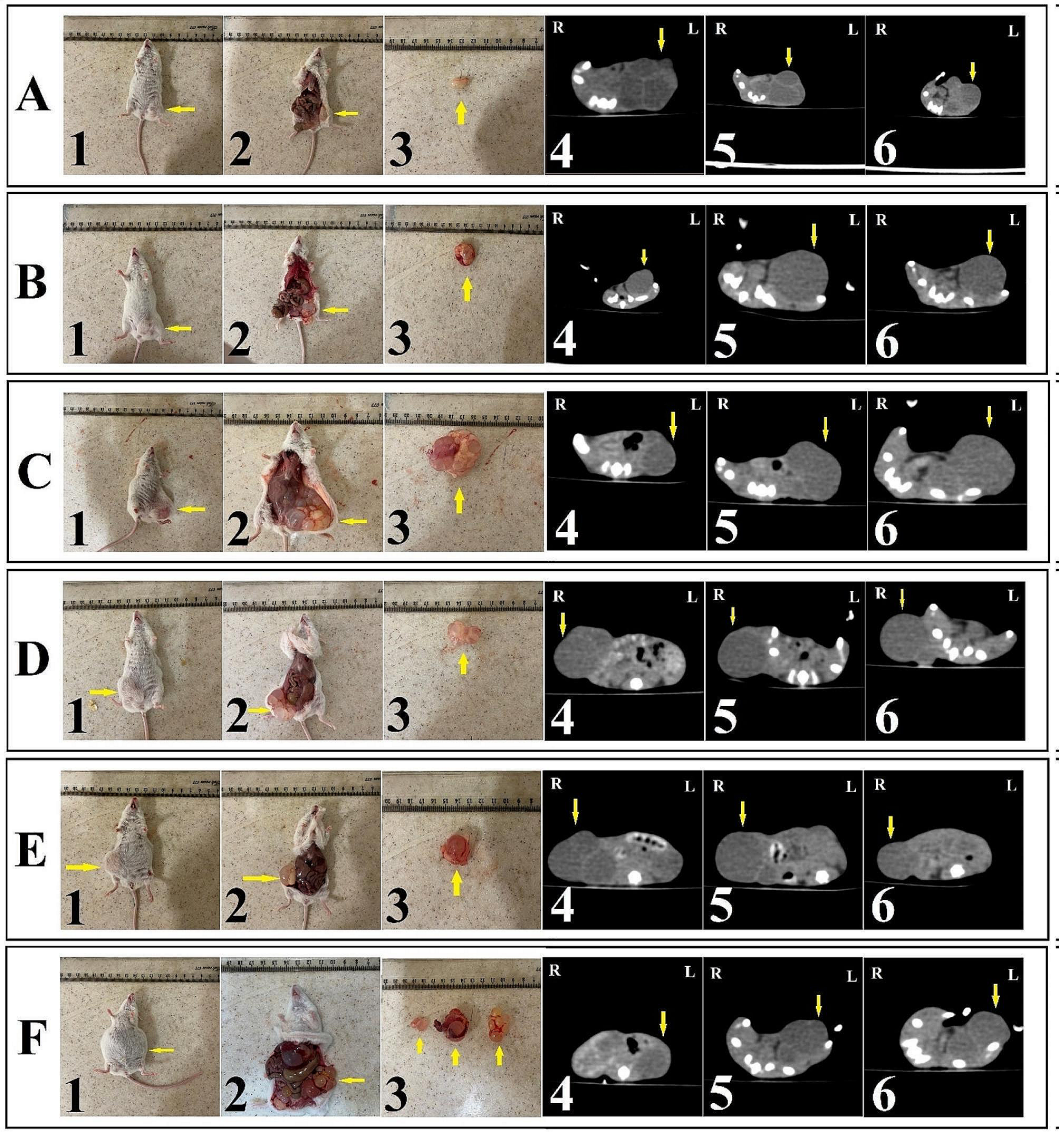
Secondary CE cyst evaluation using autopsy and CT scan in different groups. **A**: treated with curcumin nanoemulsion (CUR-NE) (40 mg/kg/day) (CEI1); **B**: treated with CUR-NE (20 mg/kg/day) (CEI2); C: treated with NE without CUR (CEI3); **D**: treated with curcumin suspension (CUR-S) (40 mg/kg/day) (CEI4); **E**: treated with ABZ as a positive control group (150 mg/kg/day) (CEI5) and **F**: received sterile PBS as negative control group (CEI6); 1, 2: gross images of mice on day 60 after treatment; 3: secondary CE cysts recovered after 60 days of treatment; 4: CT scan image before treatment; 5: CT scan image on day 30 after treatment; 6: CT scan image on day 60 after treatment; R: right side; L: left side. The boundaries of cysts are indicated by a yellow arrow

Before treatment, CT scan was used to assess secondary CE cysts, and these results were compared to CT scan results taken on 30 and 60 days after treatment (Fig. 4).

During the treatment period, two mice from the negative control group died due to excessive growth of secondary cysts, and the mortality rate in this group showed a statistically significant difference from the other study groups (*P* = 0.031). Table 2 shows the mean ± SD cyst area measurement before treatment and 60 days after treatment in different groups. The mean area of CE cysts in the CEI1, CEI4, and positive control groups decreased post treatment, while it increased in the CEI2, CEI3, and negative control groups. The mean area of CE cysts after treatment in the negative control group was significantly higher than the mean area of CE cysts before treatment (*P* = 0.033). There was no significant difference in the mean area of CE cysts between the groups (*P* = 0.862).

**Table 2:** Mean ± SD cyst area before and 60 days after treatment in different BALB/c groups

Groups	n	Cyst area (mm) using digimizer measurement		Cyst area (mm) using CT scan measurement		
		Mean ± SD before treatment	Mean ± SD after treatment	Mean ± SD before treatment	Mean ± SD after treatment	*P* value (Student’s t-test)
CEI1: treated with curcumin nanoemulsion (CUR-NE) (40 mg/kg/day)	6	Lack of access to secondary cysts	30.33 ± 21.91	25.83 ± 6.8	23.66 ± 6.25	0.063
CEI2: treated with CUR-NE (20 mg/kg/day)	6	Lack of access to secondary cysts	49.83 ± 18.11	24.83 ± 6.46	26.58 ± 8.02	0.499
CEI3: treated with NE without CUR (NE-no CUR)	6	Lack of access to secondary cysts	57.5 ± 25.28	29.5 ± 9.93	29.66 ± 10.52	0.95
CEI4: treated with curcumin suspension (CUR-S) (40 mg/kg/day)	6	Lack of access to secondary cysts	35.66 ± 20.81	27.3 ± 5.56	26.6 ± 7.17	0.75
CEI5: treated with ABZ (150 mg/kg/day) (PC^1^)	6	Lack of access to secondary cysts	50.66 ± 20.37	26.16 ± 7.74	24.58 ± 7.39	0.217
CEI6: received sterile PBS (NC^2^)	4	Lack of access to secondary cysts	67.75 ± 28.84	24.41 ± 6.64	26.41 ± 8.08	0.033^*^

ABZ: albendazole; PBS: phosphate-buffered saline

* *p* < 0.05

^1^Positive control; ^2^Negative control

**Fig. 5:**
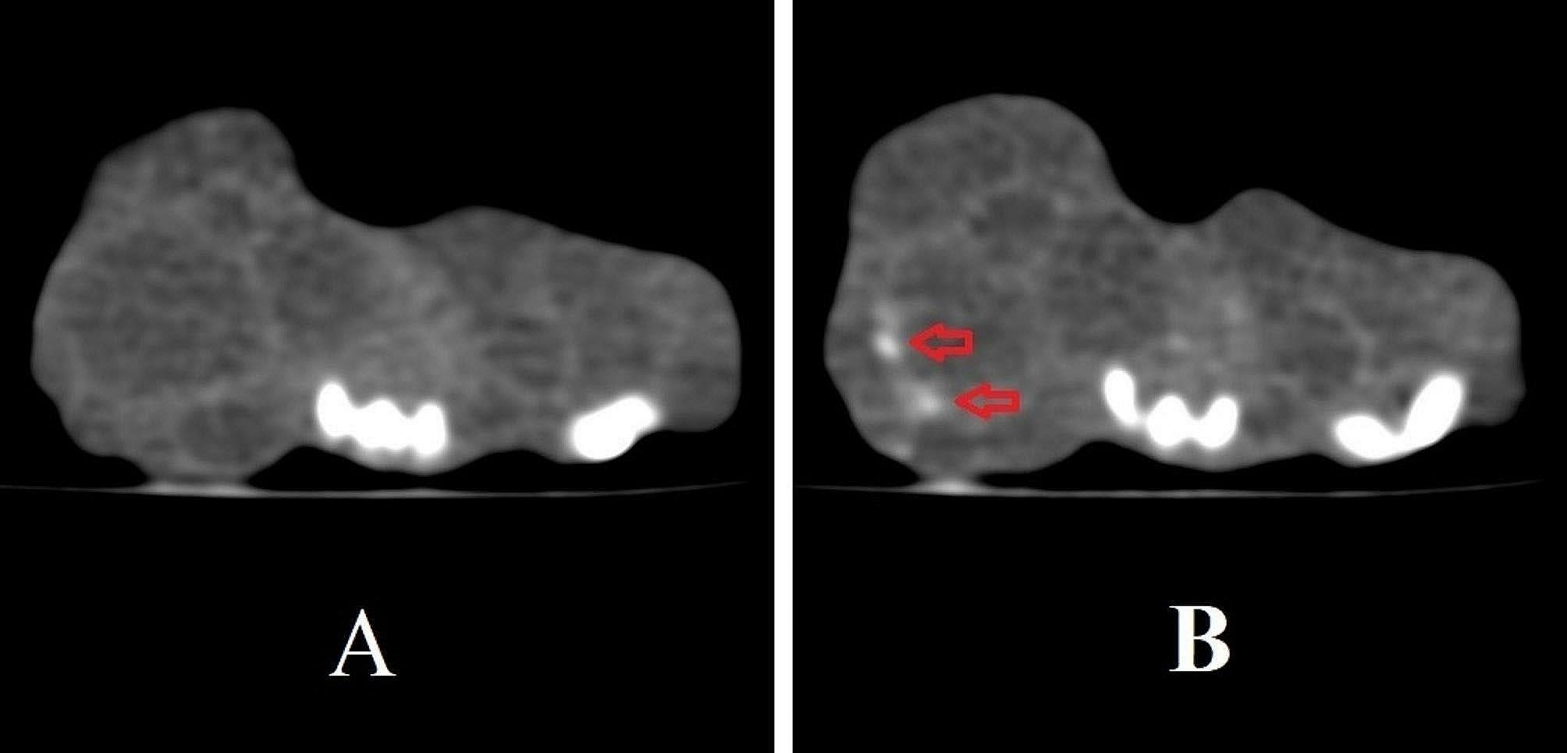
Detection of calcification using CT scan modality. **A**: Cyst in the mice treated with curcumin nanoemulsion (CEI1 group) before treatment; **B**: Cyst in the CEI1 group after 60 days of treatment. The presence of calcification in the cyst wall cavities is indicated by the red arrow

Calcification revealed by CT was not observed in the secondary CE cysts in mice before treatment. After 60 days of treatment, apparent septal calcification was observed in 40%, 16.7%, and 16.7% of mice treated with CUR-S (40 mg/kg/day), CUR-NE (40 mg/kg/day) and CUR-NE (20 mg/kg/day), respectively (Fig. 5). The difference in calcification of CE cysts before and after treatment was insignificant between the groups (*P* = 0.792).

The therapeutic efficacy was calculated for individual mice according to the specified formula. The highest therapeutic efficacy was observed in the CEI1 group, with a mean ± SD of 24.6 ± 26.89%, while the lowest was observed in the CEI3 group, with a mean ± SD of -48.03 ± 41.31% (Table 3). A significant difference was found between the treatment efficacy in different groups (*P* = 0.000). There was a statistically significant difference in treatment efficacy between the CEI1 group and the CEI3 (*P* = 0.000) and negative control groups (*P* = 0.007).

**Table 3:** Therapeutic efficacy in different BALB/c groups

Groups	n	Therapeutic efficacy (%)	
		Mean ± SD using Digimizer results	Mean ± SD using CT scan results
CEI1: treated with curcumin nanoemulsion (CUR-NE) (40 mg/kg/day)	6	55.16 ± 32.37	24.6 ± 26.89
CEI2: treated with CUR-NE (20 mg/kg/day)	6	26.34 ± 26.77	-8.19 ± 28.97
CEI3: treated with NE without CUR (NE-no CUR)	6	15.01 ± 37.34	-48.03 ± 41.31
CEI4: treated with curcumin suspension (CUR-S) (40 mg/kg/day)	6	47.28 ± 30.77	2.48 ± 12.17
CEI5: treated with ABZ (150 mg/kg/day) (PC^1^)	6	25.11 ± 30.11	8.66 ± 7.85
CEI6: received sterile PBS (NC^2^)	4	nc*	-15.49 ± 5.45

ABZ: albendazole; PBS: phosphate-buffered saline

^1^Positive control; ^2^Negative control; *Not determined

#### Determination of the therapeutic efficacy of CUR-NE using autopsy results

Digimizer software was used to calculate the area of secondary CE cysts removed from euthanized mice. The lowest mean ± SD cyst area was measured in mice of the CEI1 group (30.33 ± 21.91 mm), while the highest mean ± SD cyst area was measured in the negative control group (67.75 ± 28.84 mm) (Table 2). There was no significant difference in the area of CE cysts between groups calculated using Digimizer software (*P* = 0.172).

The therapeutic efficacy was calculated for each group using the mean cyst area measured by Digimizer software (Table 3). The highest therapeutic efficacy was observed in the CEI1 group, with a mean ± SD of 55.16 ± 32.37%, while the lowest was observed in the CEI3 group, with a mean ± SD of 15.01 ± 37.34. There was no significant difference in treatment efficacy between groups using autopsy results (*P* = 0.187).

#### Evaluation of histopathological changes in secondary CE cysts

**Fig. 6:**
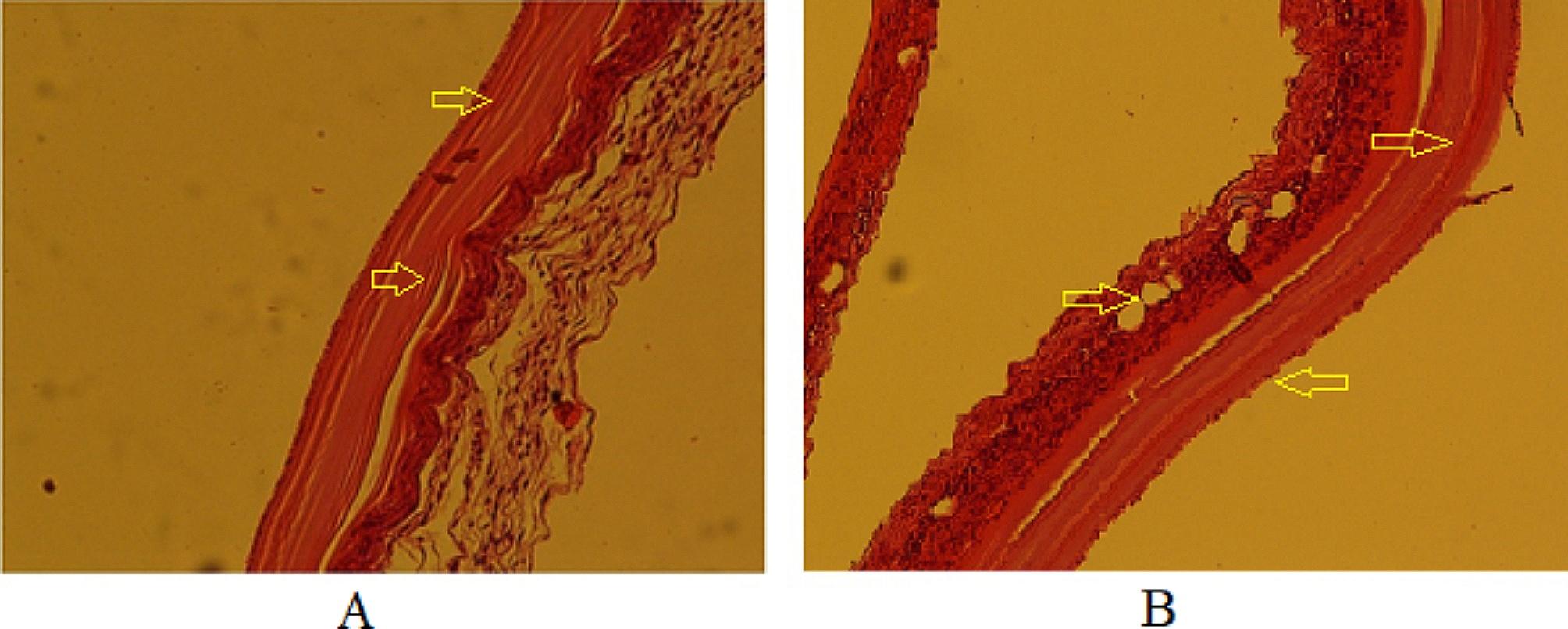
Histopathological sections of the positive control group (CEI5). **A**: Disorganized cyst wall with a thin laminated layer x100, H&E; **B**: Irregular cyst wall with a low number of cells in the germinal layer and cavitation of the adventitial layer x100, H&E

Histopathological sections were prepared from CE cysts obtained from mice. An irregular cyst wall, a thinned laminated layer, a lower number of cells in the germinal layer, and cavities in the fibrous layer (adventitial layer) were observed in the histopathological section of the CE cysts in mice belonging to the positive control group (CEI5) (Fig. 6).

**Fig. 7:**
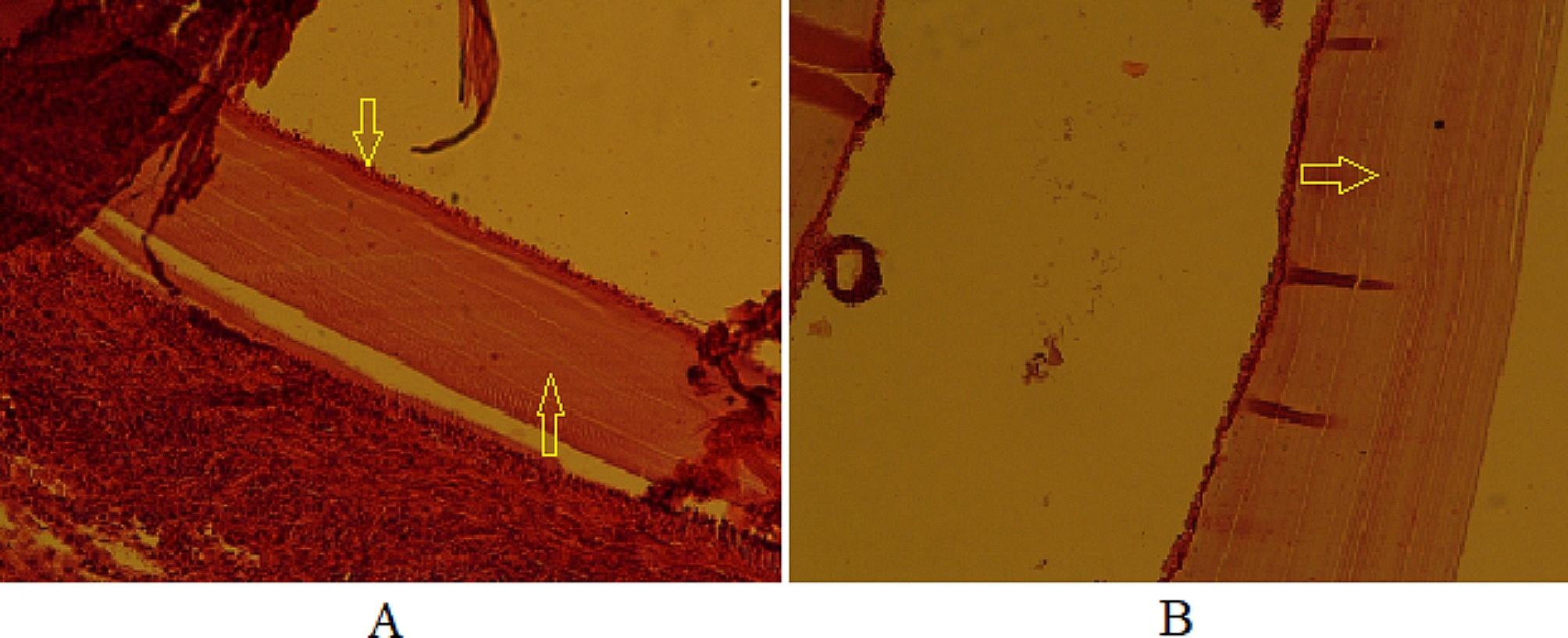
Histopathological sections of CEI6 (left image) and CEI3 (right image) groups. **A**: All layers of cyst wall are tight and regular in CEI6 group x100, H&E; **B**: similar to CEI6, no significant alteration in cyst layers was observed in CEI3 group x100, H&E

The CEI6 (negative control) and CEI3 (treated with NE-no CUR) groups had similar histopathological features, with regular and intact layers of cysts that were fully differentiated, as well as accumulation of inflammatory cells around the cyst wall (Fig. 7).

**Fig. 8:**
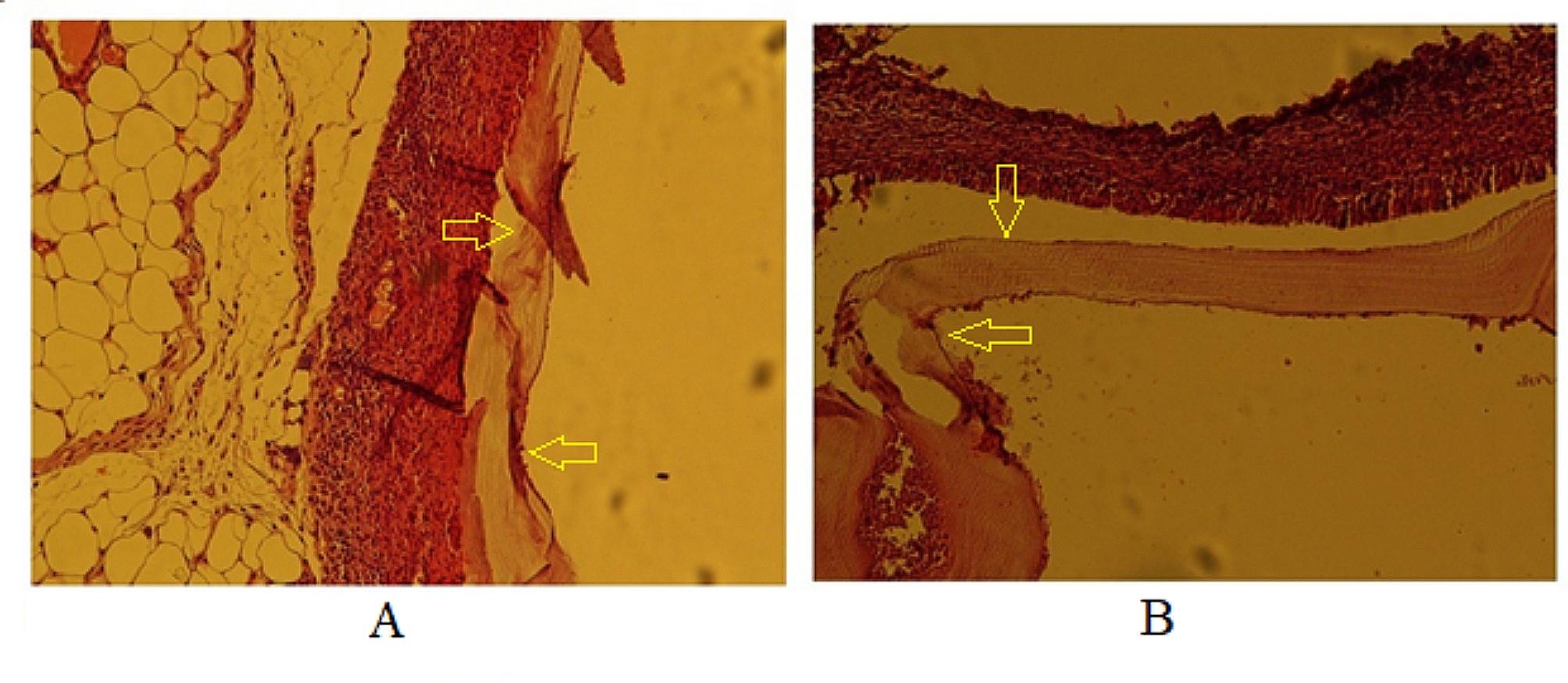
Histopathological sections of CEI2 (left image) and CEI4 (right image) groups. **A**: The cyst wall is completely degenerated and ruptured in the CEI2 group x100 H&E; **B**: The cyst wall is completely degenerated and ruptured in the CEI4 group x100 H&E

Thinning, softening, and degeneration of the cyst layers occurred in the CEI2 (treated with 20 mg/kg/day of CUR-NE) and CEI4 (treated with CUR-S) groups. In some mice in these two groups, the cyst structure was completely destroyed. The accumulation of inflammatory cells around the cysts was less observed in the CEI2 group than in the CEI4 group (Fig. 8). Despite the increase in the mean cyst area in the CEI2 group after treatment, the cyst layers were damaged according to histopathological evidence.

**Fig. 9:**
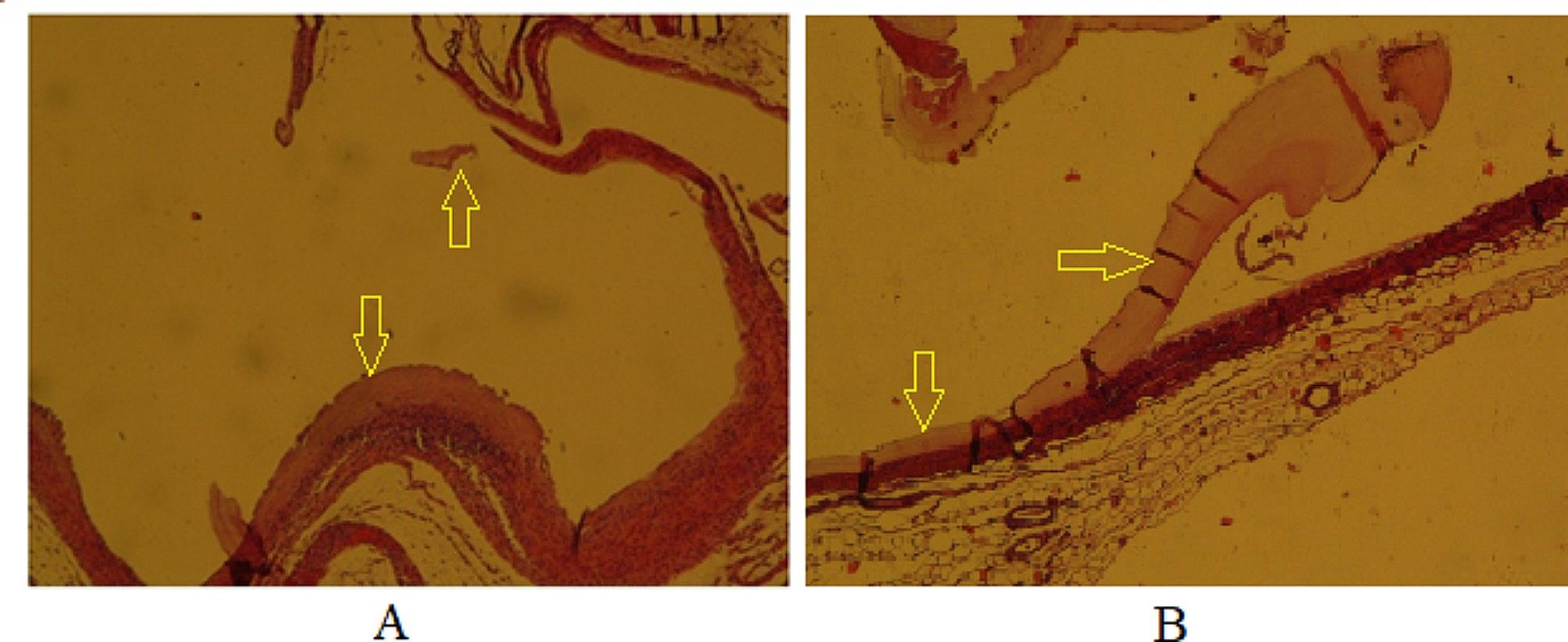
Histopathological sections of mice treated with curcumin nanoemulsion (40 mg/kg/day) (CEI1). **A**: Cyst layers disappeared x40, H&E; **B**: Cyst wall is completely degenerated, and ruptured and the only remnant of degenerated cyst is seen x40, H&E

The histological sections revealed that the cyst structure was completely destroyed in the mice of the CEI1 (treated with 40 mg/kg/day of CUR-NE) group, with only some traces of the degenerated cyst layers remaining (Fig. 9). The best histopathological result belonged to the CEI1 group.

## Discussion

The present study used CT scan modality to monitor and measure the size of the CE cyst before, during, and after treatment to avoid killing the mice before finishing the treatment protocol. CT is particularly valuable for osseous organ involvement and demonstrates the size, number, and local complications [41]. Calcifications of the cyst wall, internal septa, floating membranes and daughter vesicles are easily detected by CT [42, 43].

Different genotypes of *E. granulosus* may exhibit varying levels of host selectivity and pathogenicity [44]. Since the *E. granulosus* sensu stricto (s.s) is the predominant genotype in humans and livestock in Iran and no in vivo study has determined the genotype of PSCs before inoculation into laboratory mice; the current study, used *E. granulosus* s.s. for its protocol.

BALB/c mice are suitable breeds for inducing secondary CE infection. CE cysts are induced in more organs of the BALB/c breed compared to the NMRI and C57BL/6 breeds [45]. In the present study, CE cysts developed six months after IP injection of *E. granulosus* PSCs in inbred BALB/c mice. However, other studies have indicated that secondary cysts develop in BALB/c mice after 4–8 months [46, 47].

Treatment of CE has always been challenging due to the difficult decision between the invasiveness of surgery and the time-consuming and expensive nature of chemotherapy. For many years, surgery was the only treatment option [8]. However, currently, surgery is not always considered the first and best treatment option for CE, except in special cases [9]. Surgery has been associated with postoperative complications. However, a better prognosis has been observed when it is combined with chemotherapy (pre and/or postoperative) [48, 49]. Administration of ABZ preoperatively significantly reduced the rate of viable PSCs in patients with CE [50, 51]. In a study by Shams-Ul-Bari et al. 72 patients with CE cysts were divided into two groups. In the first group, patients underwent surgery directly, but in the second group, patients received ABZ for 12 weeks before and after surgery. The results indicated that the second group had no viable cysts at the time of surgery. The recurrence rate in the first group was 16.66%, while no recurrence was observed in the patients receiving ABZ [52]. However, ABZ has limitations (poor intestinal absorption, limited penetration from the CE cyst layers, numerous side effects, and high cost) that have driven researchers to investigate new medicines with higher efficacy and lower side effects for CE treatment [10, 14]. The current study revealed that curcumin nanoemulsion (40 mg/kg/day) was more therapeutically effective than ABZ and did not cause significant pathological effects on liver tissue in an in vivo evaluation. The use of curcumin is increasing due to its demonstrated multiple antimicrobial and anti-inflammatory effects [53–56]. However, due to its limited solubility and bioavailability, its routine form cannot be used as a therapeutic agent. The utilization of a nanoemulsion form of curcumin has led to improvements in its solubility and bioavailability and consequently its clinical application [57]. In 2018, Azami et al. used a curcumin nanoemulsion (CUR-NE) to treat toxoplasmosis in laboratory mice. They showed that this compound significantly reduced parasite load and inflammation in mice with no toxicity [58]. Investigation of the anthelmintic effects of CUR-NE on *Schistosoma mansoni* cercariae, schistosomules and adult stages, revealed optimal activity against the adult stage with decreased motor activity of the worms [59]. The protoscolicidal effects of curcumin and nanocurcumin have been shown in several studies. Tabatabaee et al. in 2022 evaluated the protoscolicidal effects of curcumin in an in vitro study. PSCs were incubated with different concentrations of curcumin (40, 80, 160, 320 µmol) for various lengths of time (30, 60, 120, 250 and 480 min). In their study, curcumin showed protoscolicidal effects only at high concentrations [27]. In 2019, the effect of *Curcuma longa* essential oil on *E. granulosus* PSCs was evaluated. The results showed that all PSCs were destroyed by curcumin essential oil in 5 min at a concentration of 200 µl/ml and in 10 min at a concentration of 100 µl/ml [25]. Another study demonstrated that the fatality rate and dimensions of *E. granulosus* PSCs were significantly affected by different concentrations of chitosan-curcumin nanoparticles [26]. The effects of CUR-NE on *E. granulosus* PSCs were investigated in an in vitro study by Teimouri et al. in 2023. They indicated that the CUR-NE concentration was inversely proportional to PSCs viability. No PSCs survived after 120 min of incubation with 1250 and 625 µl/ml CUR-NE [28]. However, its therapeutic efficacy against secondary CE has not yet been investigated in an in vivo study. The current study investigated the therapeutic efficacy of CUR-NE on secondary CE in BALB/c mice.

Various factors, such as cysts appearance and color, the weight of the mice, the size of the secondary cysts, histopathological and structural (SEM/TEM) analysis, and measurement of different parameters in serum (liver biochemical parameters, various cytokines, and oxidative stress status), were used to evaluate the secondary CE cysts in different in vivo studies after the end of treatment [60–63]. In all the mentioned studies, the size of the secondary CE cysts was evaluated by comparing the cyst size in the treated group with that in the control group. However, the current study determined the cyst size for each mouse individually by CT scan. The inability to monitor and follow-up on secondary cysts has been identified as a challenge in the in vivo study [64]. This challenge can be solved using CT scan modality. Using a CT scan, secondary CE cysts can be monitored, and their progress (changes in size and calcification) can be compared before, during and after treatment [65]. Mao et al. in 2017 determined the efficiency of CT scan modality in the identification of CE in sheep. The results indicated that CT is a suitable tool for determining the size and type of CE cysts in the liver and lungs of sheep [66]. The utilization of CT scans for detecting and characterizing CE cysts is very limited to humans and sheep. Separate measurement of CE cyst size before and after treatment in each individual mouse to assess treatment efficacy has not been performed earlier. In the present study, the mean therapeutic efficacy of CUR-NE (40 mg/kg/day) in secondary CE was reported to be 24.6% in the CEI1 group using CT scan results.

Lv et al. in 2013 reported that treatment with Huaier extract in combination with ABZ liposomes reduced germinal cells and destruction of laminated layers in secondary CE cysts in mice [67]. Zhang et al. in 2018 reported significant alterations in the germinal and laminated layers of CE cysts in mice after treatment with *Sophora moorcroftiana* alkaloids in combination with Bacillus Calmette–Guérin [62]. In the present study, the CEI1 group that received CUR-NE (40 mg/kg/day) also showed degeneration of the germinal and laminated layers. The layers of the CE cysts were fragmented, and only remnants of degenerated cysts were observed. Furthermore, a reduction in the thickness and number of germinal layer cells was observed in the positive control group. However, the best outcome was achieved in the CEI1 group.

According to Pensel et al. in 2015, the therapeutic efficacy of ABZ and ABZ-loaded lipid nanocapsules on secondary CE cysts was 46.9% and 91%, respectively. The difference in therapeutic efficacy between ABZ-loaded lipid nanocapsules and the negative control group was statistically significant (*P* < 0.01). To determine the therapeutic efficacy, the difference in the weight of secondary cysts between the untreated group and treated with ABZ and ABZ-loaded lipid nanocapsules was used [39]. In 2019, Nassef et al. reported the therapeutic efficacy of ABZ and ABZ-loaded silver nanoparticles against *E. granulosus* infection in experimental mice as 22.9% and 63.9%, respectively [68]. In both mentioned studies, the differences in cyst size or weight between the treated and untreated control groups were the measure of therapeutic efficacy, since the cyst size in individual mice was unknown before treatment. In the present study, in addition to determining the therapeutic efficacy of CUR-NE using a CT scan, the therapeutic efficacy of this compound was also determined by measurement criteria of secondary cysts of euthanized mice after autopsy. In this section, our work was similar to that of Pensel and Nassef. The mean therapeutic efficacy of CUR-NE at 40 mg/kg/day and 20 mg/kg/day was 55.16% and 26.34%, respectively. The method of calculating therapeutic efficacy can significantly affect the results. When the size of secondary CE cysts before treatment is available, it is possible to determine the therapeutic efficacy for each mouse individually, and the result is markedly different from when each mouse is compared with the control group.

Commonly, cyst death is marked by an important sign, which is cyst wall calcification. There are a few data on the presence of vesicles or PSCs in calcified cysts, which are usually considered an index of cyst death and are believed to be an indicator of cyst inactivity [69]. The potential importance of calcification in relation to CE cyst activity has been evaluated in the study done in China. This study revealed the correlation of genes (biomarkers) with calcification by analyzing the expression of galecitin-4 (LGALS4) and acid ceramidase (ASAH1) in patients with calcified and noncalcified cysts [70]. The immune response may contribute to parasite death, but the exact mechanism of cyst degeneration is unclear [69]. Calcification may be initiated by the Th1 response, which is usually harmful to the parasite, through macrophages that may produce osteopontin, a potent regulator of calcium deposition in tissue [71]. Several studies have reported that CE cysts with calcification are not limited to inactive cysts (type CE4 or CE5) and may be observed in all stages of the cysts [72–74]. However, the expansion of calcification was clearly greater in inactive cysts, and the frequency of calcification increased with the progression of the degenerative process of the CE cysts. Evaluation of calcification along with performing immunological tests, particularly IgG4 and IgE may help to define cyst activity better [69]. In the study of Hosch et al. a high proportion of calcification in the peripheral wall of human CE cysts was reported (approximately 50% of all evaluated cysts) [73]. When radiography examines human CE cysts, calcification in the pericyst appears as a curvilinear or ring-like pattern in 20–30% of cases [75]. In the current study, cyst calcification was not detected in the CT scan of secondary CE cysts before treatment. Nevertheless, after treatment, obvious cyst calcification was observed in the CT scans of 40%, 16.7% and 16.7% of mice in the CEI4, CEI1, and CEI2 groups, respectively. CT is the modality of choice for detecting calcifications, alone or together with other methods may be an essential feature of diagnosis and treatment evaluation [76–78]. So, measuring and comparing the dimensions of secondary cysts before and after treatment could be a major criterion to assess the therapeutic efficacy in a CE-infected mouse model.

## Conclusion

The current study showed a significant therapeutic effect of CUR-NE (40 mg/kg/day) on secondary CE cysts in mice. An apparent septal calcification of several cysts revealed by CT scan and the destructive effect on CE cysts observed in histopathology are two critical key factors that suggest curcumin compounds, especially their nanoemulsion form, could be a potential treatment for cystic echinococcosis. The employment of nanotechnology may offer a safe, effective and cheap remedy.

## Data Availability

All data generated or analyzed during this study are included in this published article. The raw data are available from the corresponding author upon reasonable request.

## Abbreviations

CUR: Curcumin
NE: Nanoemulsion
CUR-NE: Curcumin nanoemulsion
CUR-S: Curcumin suspension
NE-no CUR: Nanoemulsion without Curcumin
ABZ: Albendazole
*E. granulosus*: *Echinococcus granulosus*
PSCs: Protoscoleces
CE: Cystic echinococcosis
CEI: Cystic echinococcosis infected
CENI: Cystic echinococcosis noninfected
CT: Computed tomography
WHO: World Health Organization
mm: Millimeters
DMEM: Dulbecco’s Modified Eagle’s Medium
FBS: Fetal bovine serum
MTT: 3-(4,5-dimethylthiazol2-yl)-2,5-diphenyltetrazolium bromide
OD: Optical density
PCR: Polymerase chain reaction
IP: Intraperitoneally
H&E: Hematoxylin and Eosin
Bp: Base pair
s.s: sensu stricto
ESR: Erythrocyte sedimentation rate
LGALS4: Galecitin-4
ASAH1: Acid ceramidase
